# Bacterial Community Composition and Isolation of Actinobacteria from the Soil of Flaming Mountain in Xinjiang, China

**DOI:** 10.3390/microorganisms11020489

**Published:** 2023-02-16

**Authors:** Zixuan He, Yuxian Wang, Xiaoyu Bai, Min Chu, Yuanyang Yi, Jing Zhu, Meiying Gu, Ling Jiang, Zhidong Zhang

**Affiliations:** 1 College of Life Sciences and Technology, Xinjiang University, Urumqi 830046, China; 2 Xinjiang Key Laboratory of Special Environmental Microbiology, Institute of Microbiology, Xinjiang Academy of Agricultural Sciences, Urumqi 830091, China; 3 State Key Laboratory of Materials-Oriented Chemical Engineering, College of Food Science and Light Industry, Nanjing Tech University, Nanjing 211816, China; 4 College of Life Sciences, Xinjiang Normal University, Urumqi 830054, China

**Keywords:** Flaming Mountain, bacterial community composition, actinobacteria, isolation and identification, function evaluation

## Abstract

In this work, bacterial community composition and actinobacteria resources were explored in extremely hot and hyper-arid areas of Flaming Mountain. This was achieved through a combination of PCR amplicon sequencing of bacterial 16S rRNA gene and cultivation-dependent isolation and characterization efforts. According to the high-throughput sequencing results and soil characteristics, 11 kinds of media were firstly designed to isolate actinobacteria, following the screening and identification of related strains. The results showed that a total of 2994 operational taxonomic units (OTUs) were obtained, involving 22 phyla, 77 orders and 121 genera. Among them, actinobacteria with the relative abundance of 8% ranked third, accounting for 33 genera and 47 species. A total of 132 strains distributed by eight families and 11 genera of actinobacteria were isolated from 11 media, of which six strains were potential new species. Furthermore, the functional characteristics of isolated strains were preliminarily evaluated. The results showed that the obtained strains generally had tolerance against heat, salt and alkali. Fifty-two strains had antibacterial activity, 69 strains could produce hydrolases, and 12.4% of the strains had quorum sensing inhibitory activity. The present study has laid a solid foundation for further understanding the bacterial diversity and exploiting actinobacteria resources in the Flaming Mountain area.

## 1. Introduction

Actinobacteria are biotechnologically valuable as important sources of diverse natural products. In fact, more than 30,000 natural products have been isolated and identified from microorganisms, and more than 50% of bioactive compounds were derived from actinobacteria, including antibiotics, anticancer drugs, immunosuppressive drugs, enzymes, enzyme inhibitors, and other therapeutic or bioactive compounds [1]. However, it is increasingly difficult to discover novel bioactive compounds from actinobacteria in normal environments [2]. Consequently, it is necessary to search for undiscovered new species from unusual habitats. A recent review illustrated that abundant novel compounds were produced by microbes isolated from extreme habitats [3]. Desert environments are often extremely harsh, with high or low temperatures, poor nutrients, very few organic carbon sources, high levels of UV radiation, extreme pH or salinity, and other abiotic stresses [4]. However, recent studies suggested that desert ecosystems are an unexplored source of new extremophiles with high tolerance to extreme conditions. In deserts, actinobacteria are among the most frequent groups, generally accounting for over 35% of all microorganisms. A study showed that the diversity of the actinobacterial community increased with the increase in drought degree, as well as the proportion of potential new species [5]. In Chile’s Atacama Desert, the driest desert on Earth, actinobacteria are widespread and frequently dominate prokaryotic communities (75–98%) [6]. The most explored desert soil sample contained more than 92% new actinobacteria species. Therefore, the extreme conditions of the desert provide a strong selective pressure that shapes the diversity of actinobacterial communities.

Xinjiang has a vast territory with diverse ecological habitats, more than 80% of which is covered by the Gobi Desert. A large number of novel actinobacteria were isolated and identified from these unusual habitats. Recently, a novel Actinobacterium designated EGI 80759T was isolated from Karamay and was proposed to be a representative of a novel species in the novel order *Egibacterales* [7], which is the highest taxonomic unit named by Chinese microbial taxonomists. The Flaming Mountain region is located on the northern edge of the Turpan Basin, known as “Fire State” or “Land of Fire” [8]. It is extremely arid with very hot summers, very cold winters, and minimal precipitation, which amounts to only 20 mm per year. Few studies have investigated the microbial community composition of Flaming Mountain region, but it was reported that it contains fewer microbes than other parts of the Gobi Desert or other deserts, and bacteria were the most abundant [9]. In a study of microorganisms in the Turpan depression, 39 strains of actinobacteria were isolated [10] and showed strong antibacterial activity against *Verticillium dahlia*, the pathogen causing verticillium wilt. In addition, two novel genera in the family of *Pseudonocadiaceae* and one new species of *Streptomyces* were named. Overall, this previous study suggested rich diversity of actinobacteria in the desert environment.

In the present study, the bacterial community composition of soils from Flaming Mountain was analyzed by high-throughput sequencing. Actinobacteria were isolated from 11 different media, nine of which were recommended by the Known Media Database (KO-MODO) (http://komodo.modelseed.org/default.htm) (accessed on 23 August 2021) [11], and two were designed based on soil characteristics. We compared the community composition of culturable and uncultured actinobacteria and further investigated their growth characteristics and functions. This study can provide a basis for further revealing the diversity of microbial communities and mining valuable actinobacterial resources in the extremely hot and arid environment of the Gobi Desert.

## 2. Materials and Methods

### 2.1. Sample Collection and Physicochemical Characteristics of Soils

**Figure 1:**
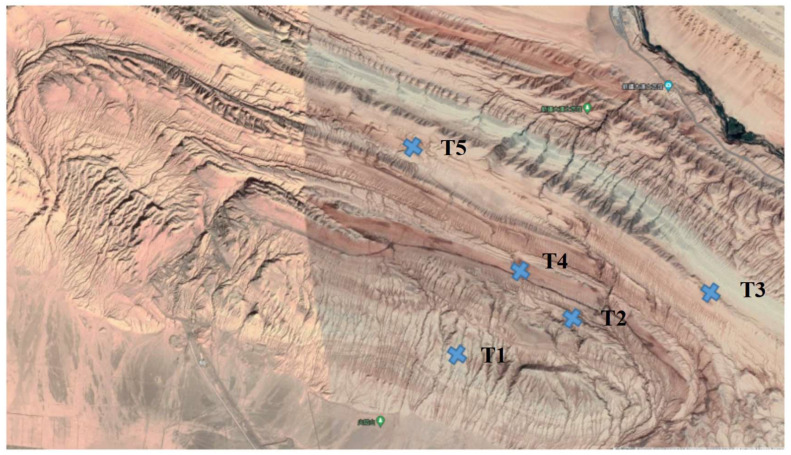
Location of sampling points in Flaming Mountain area (soil samples marked in blue).

Soil samples were collected from Flaming Mountain in Xinjiang in July 2021 (31–43 °C) (Figure 1). A few famous tourist attractions in Turpan surround the Flaming Mountain, so we tried our best to select sample sites in an unfrequented area that is a typical hyper-arid habitat. Five sites were selected approximately 1 km apart in a circle with a diameter of 3 km. Three samples were taken randomly from a 5 × 5 m homogeneous area at the site. All samples (about 1 kg each) were taken at a depth of 10–15 cm from the ground surface using a shovel. After removing impurities, the sample was thoroughly mixed, and 2 kg was obtained by the quartering method [12], placed in sterile polypropylene bags, and stored at 4 °C until analysis. All of the samples were analyzed for soil moisture, pH, soluble salt, total nitrogen and organic matter (Table 1). Soil moisture was determined gravimetrically by drying the soil samples for 24 h at 105 °C (expressed as percentage of dry weight) [5]. Soil pH was determined in a 1:2.5 (*w/v*) aqueous solution using a conventional pH meter [13], and other physicochemical parameters of the soil samples were determined according to the soil agrochemical analysis method [14].

### 2.2. DNA Extraction and High-Throughput Sequencing

The MP FastDNA 50 mL Spin Kit for Soil (MP Biomedicals, Santa Ana, CA, USA) was used to extract genomic DNA of the samples. A maximum of 10 g of soil was used in each 50 mL Garnet Lysing Matrix tube. Due to the low microbial content, 200 g soil samples were divided into 20 portions and extracted simultaneously, following the manufacturer’s instructions. All extracted DNA was combined and then eluted by adding 1 mL of TES. Finally, agarose gel electrophoresis was used to assess the purity and concentration of the extracted DNA. The diluted genomic DNA was used as the template to amplify the bacterial flora of the sample using the universal primers 27F and 1492R, and the DNA information of the amplified products in the V3–V4 region of 16S rDNA was identified [15].

Total DNA was used as the template for PCR, and the V3 to V4 hypervariable regions of 16S rRNA genes were amplified using the universal bacterial primers 5′-ACTCCTACGGGAGGCAGCAG-3′ (338F) and 5′-GGACTACHVGGGTWTCTAAT-3′ (806R). The Illumina PE100 high-throughput sequencing platform of Novo Source was used for sequencing. The QIIME (Version 1.9.1) software package [16] was used to demultiplex and quality-filter raw reads by removing reads meeting the following criteria. The data were processed using the High Performance Computing platform and analyzed to obtain the available sequences [17]. Using the sequences of each sample as the base unit, all effective tags of all samples were clustered using the Uparse algorithm [18], and by default, the sequences were clustered into OTUs with 97% agreement. A fast multiple sequence comparison was performed using MUSCLE (Version 3.8.31) [19] software to obtain the phylogenetic relationships of all representative OTU sequences.

### 2.3. Microbial Culture and Isolation of Actinobacteria

Microbes were detected using the serial dilution method and the spread plate technique. Aliquots comprising 5 g of each soil sample were suspended in 50 mL of sterile physiological saline (0.85%, *w/v*), shaken at room temperature for 30 min, and then serially diluted in saline solution. Then, 100 μL of the dilutions were spread onto PDA plates with chloramphenicol, NA and GAUZE’s medium for fungal, bacterial and actinobacterial counting. Actinobacteria were isolated using 11 different media. Among them, nine media (GAUZE’s, M2, M3, M6, M9, 214, R2A, 656, SSY, MA and GSM medium) were recommended by the Known Media Database (KOMODO) (http://komodo.modelseed.org/default.htm) (accessed on 23 August 2021) based on the 16S rDNA high-throughput sequencing results, while MA and R2A media were selected based on soil characteristics (Supplementary Table S1).

Aliquots comprising 5 g of each soil sample were suspended in 50 mL of sterile physiological saline (0.85%, *w/v*), shaken at room temperature for 2 h, and then serially diluted in saline solution. Culture isolation was performed by placing 100 μL of the dilutions (10^−1^) onto each of the isolation media mentioned above. All of the plates were incubated at 40 °C for 7 days. The strains were then further purified on the original medium from which the strain was isolated. The plates were incubated at 40 °C for 5–30 days, and the colonies were observed periodically. Pure cultures were obtained after successive rounds of sub-culturing, ISP2 and original isolation medium were used to preserve strains. The cultures were preserved on their respective media slants at 4 °C and 30% glycerol at − 80 °C.

### 2.4. The 16S rRNA Gene Sequencing and Sequencing Data Processing

The 16S rRNA gene sequencing and determination of a reasonable size sequence was performed as described before [20]. Briefly, DNA was extracted using the TIANamp Bacteria DNA Kit (Tiangen Biochemical, Beijing, China), following the manufacturer’s instructions. DNA was eluted in 100 µL of elution buffer. Amplification from the genomic DNA samples was performed using the eubacterial universal primers 27F 5′-AGAGTTTGATCCTGGCTCAG-3′ and 1492R 5′-GGTTACCTTGTTACGACTT-3′. Amplification reactions were set up in a final volume of 30 µL. The amplification conditions were as follows: pre-denaturation at 94 °C for 5 min, followed by 30 cycles of denaturation at 94 °C for 30 s, annealing at 55 °C for 30 s and extension at 72 °C for 45 s, and followed by a final extension at 72 °C for 20 min. The amplified PCR products were sent to Shanghai Bioengineering Technology Company. These gene sequencing results were then aligned with all available 16S rRNA gene sequences of validly described species using the EZbioCloud 16S database (www.ezbiocloud.net) and NCBI (www.blast.ncbi.nlm.nih.gov) to reveal approximate phylogenetic relationships.

### 2.5. Strain Characteristics

The growth of all strains at various NaCl concentrations (5,10, 12 and 15% *w/v*), and different temperatures (20, 45, 50 °C) was examined on ISP2 agar plates, while the pH range for growth (pH 5.0–13.0, at intervals of 1 pH unit) was assessed in ISP2 broth prepared using the buffer system as described by Fang et al. [21] The strains were incubated for 72 h, and the upper limit of tolerance of the strains was recorded. The experiments were performed in triplicate for each group. The actinobacteria were inoculated onto the enzyme activity screening medium by Spot vaccination and incubated at 37 °C for 1–7 days. The activities of protease and cellulase were quantified by measuring the transparent zone around the colonies. Amylase activity was screened using 1% iodine solution to observe the discoloration of the culture medium around the colonies. The isolates were examined for their ability to inhibit the growth of wild type strains of *Candida albicans*, *Staphylococcus aureus*, *Escherichia coli*, *Pseudomonas fluorescens* and *Alternaria alternata* using the agar diffusion assay [22]. The purified strains were cultured in liquid medium at 37 °C for 3–5 days, and then 100 μL of sterile filtrate of the strains was added into wells of the indicated plates. The inhibition zones were observed after incubation at 37 °C for 3–5 days.

The strains were seeded into liquid GAUZE’s medium and incubated at 30 °C for 7 days. The fermentation broth was centrifuged and filtered through a 0.22 μm pore-size membrane. The filtrate was stored at −20 °C. *Chromobacterium violaceum* strain CV026 was added into LB liquid medium at a 1% inoculation ratio. After 16 h, 1 mL of CV026 filtrate and 20 μL C6-HSL were mixed with 25 mL of LB medium. A 9 mm well was punched, filled with 100 μL of sterile filtrate, and incubated at 30 °C for 24 h to observe the inhibition zone.

## 3. Results

### 3.1. Physicochemical and Bacteriological Characteristics of Flaming Mountain Soils

The physicochemical properties of samples from five different sites across Flaming Mountain were analyzed. All samples were alkaline (pH from 8.74 to 9.08), and had a high soluble salt content (668.4–219.6 g/kg), as well as low organic matter and nitrogen contents. The water content was only 0.258%. In general, the soil characteristics reflected the extremely arid, high salinity and low nutrient environment of the desert. This largely explains the low microbial counts in the Turpan area (Table 2). The average microbial count in the collected samples was 4.27 × 10^3^ CFU/g. T2 had the lowest microbial count, reaching only 2.1 × 10^3^ CFU/g, while T3 had the highest microbial count, at 7.88 × 10^3^ CFU/g. Actinobacteria were dominant in the samples (71~82%), indicating that Flaming Mountain has rich resources of this group of microorganisms.

### 3.2. Analysis of Bacterial and Actinobacterial Community Structures by High-Throughput Sequencing

A total of 1,280,671 original bacterial 16S rDNA sequences were obtained by high-throughput sequencing, 1,254,224 quality control sequences were obtained by filtering low-quality and short-length sequences, and 963,463 effective sequences were obtained by redundancy processing. The number of effective sequences ranged from 188,077 to 199,780. Finally, a total of 2994 OTUs were obtained (Table 3).

**Figure 2:**
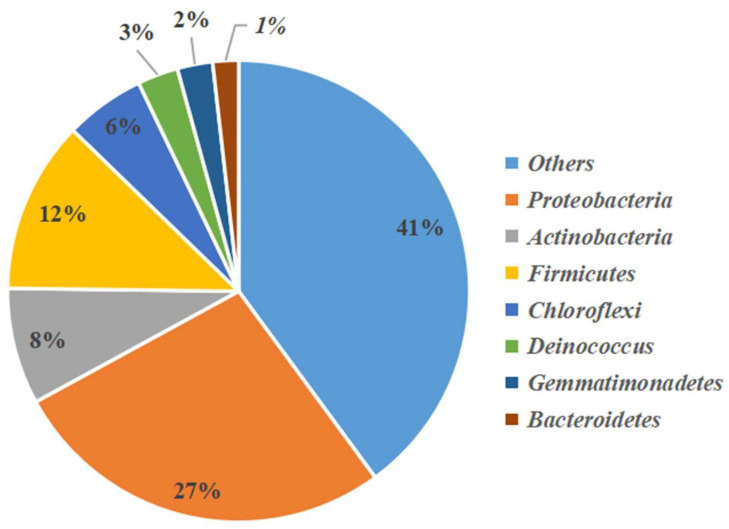
Pie chart showing the percentage relative abundance of the phyla in bacterial communities from samples analyzed by high-throughput sequencing.

According to the analysis of the high-throughput sequencing results, a total of 22 phyla, 77 orders, and 121 genera were identified in the Flaming Mountain samples. The major phyla were Proteobacteria, Actinobacteria, Firmicutes, Chloroflexi, Deinococcus-Thermus, Gemmatimonadetes and Bacteroidetes, accounting for 60% of all sequences. Proteobacteria (27%) dominated the bacterial communities in this hyper-arid area, followed by Firmicutes (12%) and Actinobacteria (8%) (Figure 2).

**Figure 3:**
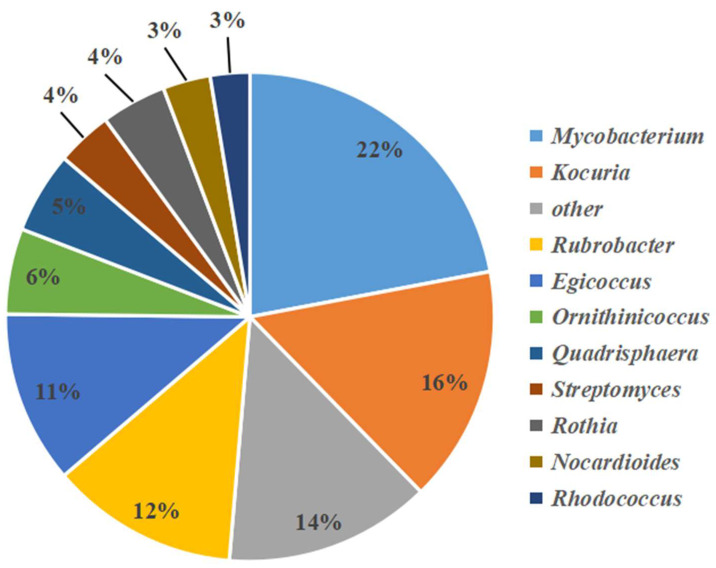
Pie chart showing the percentage relative abundance of actinobacteria genera from samples analyzed by high-throughput sequencing.

Among these, 33 genera of actinobacteria were identified, 21 genera of which had a population abundance of less than 0.1%. The 10 dominant actinobacterial genera accounting for 86% were *Streptomyces*, *Rubrobacter*, *Mycobacterium*, *Rhodococcus*, *Nocardiopsis*, *Egicoccus*, *Ornithinicoccus*, *Kocuria*, *Rothia*, and *Quadrisphaera*. Among them, the proportion of rare actinobacteria was 96%, and *Mycobacterium* was the most abundant genus (22%). A few *Streptomyces* OTUs were detected (4%) (Figure 3).

### 3.3. Analysis of Culturable Actinobacteria Diversity

#### 3.3.1. Screening and Identification of Culturable Actinobacteria

**Figure 4:**
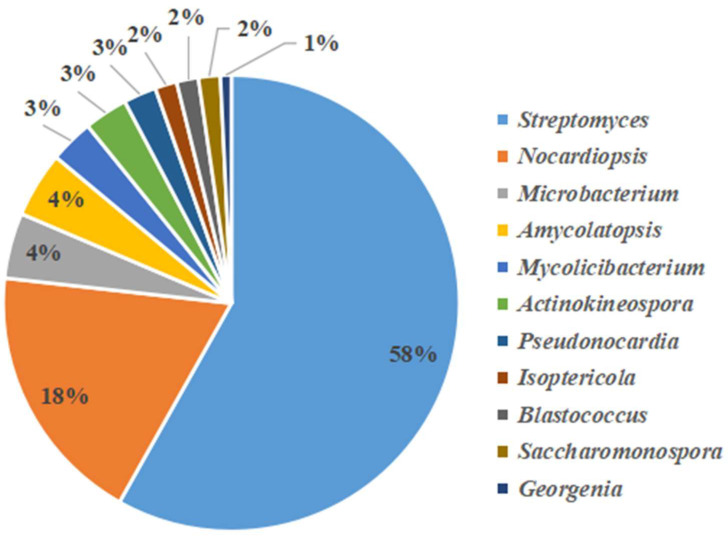
Pie chart showing the percentage relative abundance of the genera in actinobacteria from samples analyzed by the culturable method.

A total of 132 actinobacteria strains were isolated and identified according to differences in colony morphology (Figure 4). The results of 16S rRNA gene sequencing indicated that the strains belonged to eight families and 11 genera. The genera included *Streptomyces*, *Nocardiopsis*, *Actinokineospora*, *Amycolatopsis*, *Pseudonocardia*, *Blastococcus*, *Microbacterium*, *Mycobacterium*, *Isoptericola*, *Saccharomonospora* and *Georgenia*. Among them, *Streptomyces* was the primary cultivated genus, accounting for 58% of all isolates, followed by *Nocardiopsis*, which accounted for 19% (Table 4). 

#### 3.3.2. Effects of Different Media on the Screening of Actinobacteria

**Figure 5:**
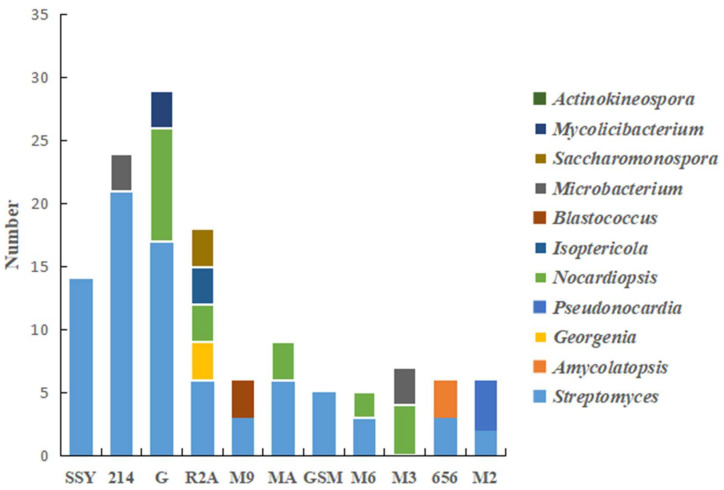
Composition and relative abundance of actinobacteria in different culture media.

Statistical analysis indicated that among the optimized media designed based on the results of high-throughput sequencing, the GAUZE’s medium yielded the most actinobacteria, including 17 strains of Streptomyces, nine strains of Nocardiopsis and three strains of Mycobacterium, followed by 214 medium, from which Streptomyces and Actinokineospora were isolated. R2A medium had better selectivity for rare actinobacteria including Georgenia, Nocardiopsis, Isoptericola and Saccharomonospora. In addition, the rare actinobacteria including Pseudonocardia, Nocardiopsis, Microbacterium and Blastococcus were also isolated on media M2, M3 and M9 (Figure 5).

#### 3.3.3. Potential New Species of Actinobacteria

Among the 132 strains, six were potential novel bacterial species with a 16S rRNA gene similarity < 98.65% [23] (Table 5). These included three strains of Streptomyces, one strain of Nocardiopsis, one strain of Georgenia and one strain of Saccharomonospora. The results preliminarily indicated that there are potential new microbial species resources in the Turpan Desert. The final taxonomic status of these strains needs to be confirmed based on polyphasic taxonomic analysis in future studies.

### 3.4. Stress Tolerance and Biological Activity of the Strains

#### 3.4.1. Stress Tolerance of the Strains

**Figure 6:**
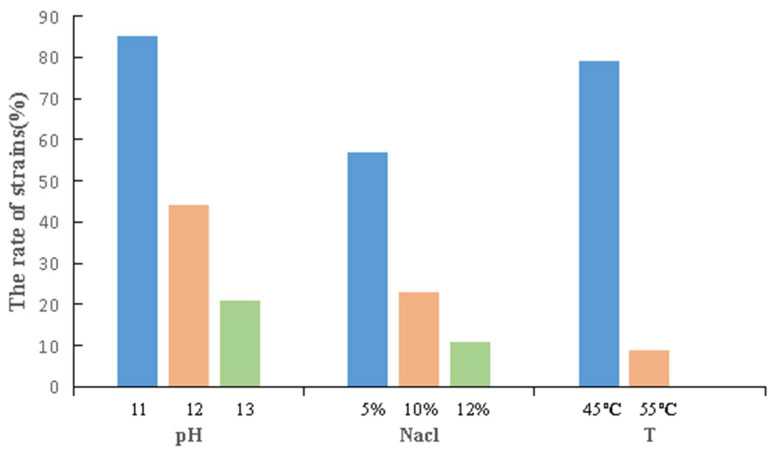
Resistance characteristics of actinobacteria.

Analysis of the stress tolerance of the isolated actinobacteria showed that most of the strains had strong stress resistance, with 57% being able to grow at 5% NaCl and 85% being able to grow at pH 11.0. In addition, 79% of the strains could grow at 45 °C, and 9% could even grow at 55 °C (Figure 6).

Some strains showed notable stress resistance (Table 6). For example, potential new species G86, G24 and GH22 had strong alkali resistance and could grow at pH 12.0, while G86 had the strongest salt tolerance and could grow at 12% NaCl. These three strains could also grow at 45 °C. Moreover, strains G131 and G114 not only had strong tolerance to salt and alkali, but could also even grow at 55 °C.

#### 3.4.2. Biological Activity of the Strains

The amylase, protease and cellulase production capacity of the 132 selected strains was analyzed by the plate transparent zone or fading ring method. The results showed that 45% of the experimental strains had amylase activity, 52.2% had protease activity and 12% had cellulase activity.

**Figure 7:**
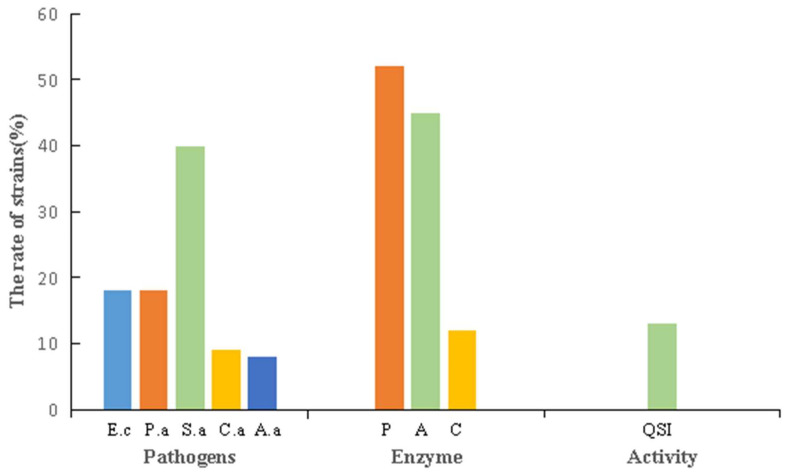
Bioactive potential of actinobacteria (E.c is *Escherichia coli*; P.a is *Pseudomonas aeruginosa*; S.a is *Staphylococcus aureus*; C.a is *Candida albicans*; A.a is *Alternaria alternata*; P is Protease; A is Amylase; C is Cellulase; QSI is QSI activity).

The antibacterial and antifungal activities of the isolates were investigated by co-culture with *Escherichia coli*, *Pseudomonas aeruginosa*, *Staphylococcus aureus*, *Candida albicans*, and *Alternaria alternata*. All 132 actinobacteria isolates from Flaming Mountain were screened for antimicrobial activities. Among them, 18% of the isolates exhibited antimicrobial activity against *Escherichia coli*, 18% against *Pseudomonas aeruginosa*, 40% against *Staphylococcus aureus*, 9% against *Candida albicans*, and 8% against *Alternaria alternata*. In addition, 13% exhibited inhibitory activity against quorum sensing (Figure 7).

Some of the strains exhibited strong biological activity (Table 7). For example, the potential new species G86 and GH22 had not only protease and amylase activities, but also antibacterial activity against Staphylococcus aureus. D119 produced three kinds of hydrolases, while also inhibiting Staphylococcus aureus and Pseudomonas aeruginosa. G114 was able to fight against Staphylococcus aureus and had a strong inhibitory effect against Alternaria alternata. Similarly, G24 could produce amylase, while also exhibiting antibacterial and even quorum sensing inhibition activities.

## 4. Discussion

Hyper-arid areas are defined by a mean annual rainfall (MAR)/mean annual evaporation (MAE) of less than 0.05 [24] or soil water content of less than 2% [25]. Hyper-arid deserts are characterized by extreme desiccation, low water activity as a consequence of high salt content, high UV irradiation, high pH and high concentrations of heavy metals [26]. Such conditions are challenging to all organisms, and these areas were once considered to be “exclusion zones”. However, recent studies found that there is abundant microbial diversity in arid and hyper-arid environments. The Atacama Desert in northern Chile, the most hyper-arid area of the world, has a MAR/MAE ratio of less than 0.05 [27]. However, there are still about 10^3^ CFU/g microorganisms in the soil (94% of which are actinobacteria), which have been invariably assigned to new species as exemplified by *Lechevalieria* and *Streptomyces* species [28]. Related studies also showed that actinobacteria were the main phylum in arid areas, and with the increase in drought degree, the relative abundance of actinobacteria increased. Actinobacteria accounted for 35 and 29% of microorganisms in samples from the Badain Jaran Desert (MAR < 40 mm) and Tenger Desert (MAR = 102.9 mm), respectively [29]. At the same time, transcriptomic studies further proved that actinobacteria maintained their life activities under hyper-arid conditions. In the Namib Desert of Namibia (temperature 21–55 °C, humidity 13–27%), a diverse and active community is dominated by actinobacteria. In this area, chemoautotrophy is an important alternative to photosynthesis for carbon cycling in desiccated desert soils, suggested that the main driving force for the change in soil microbial community activity is the acquisition of nutrients, not the environmental conditions [30]. Turpan Flaming Mountain of Xinjiang has a unique geographical location and basin topography. The region is hot and dry, with large temperature differences between day and night as well as soil desertification. The highest temperature in summer is as high as 47.6 °C, the highest surface temperature is as high as 89 °C, and the MAR/MAE is only 0.005 [31], which makes it the hottest and most hyper-arid place in China. Previous studies have shown that the species distribution of soil microorganisms in the Flaming Mountain area is dominated by bacteria, followed by actinobacteria [8]. However, there are few studies on the composition and abundance of related communities in this region, which need to be studied further.

To study bacterial community composition and isolate actinobacteria, five typical samples were collected from Flaming Mountain. The results showed that the microbial count (2.1–7.88 × 10^3^ CFU) is comparable to that of the Atacama Desert, but significantly less than that of other deserts. High-throughput sequencing yielded 2994 OTUs, encompassing 22 phyla, 77 orders and 121 genera. Among them, Proteobacteria (27%), Firmicutes (12%) and Actinobacteria (8%) were the dominant phyla. The proportion of actinobacteria was much lower than in other deserts. Related studies have shown that different extraction methods, primer lengths and annealing temperatures can lead to results with high-throughput sequencing analysis bias. When extracting total DNA from rhizosphere soil samples, it was found that different extraction methods resulted in great differences in the total DNA content and purity [32]. In addition, different primer lengths will also affect the abundance of OTUs. The community structures of the libraries created using different primer combinations were clearly different, especially the relative abundance of OTUs of some rare actinobacteria. Annealing temperature can also affect the effective reading of OTUs through primers, especially for the genus *Bifidobacterium* [33]. Therefore, the method used for extracting the total DNA from desert soil still needs to be further optimized. Thus, the populations of each sample were not analyzed in detail in this study.

High-throughput sequencing identified 47 species, 33 genera and 25 families of actinobacteria. Among them, *Streptomyces* accounted for only approximately 4%, while rare actinobacteria accounted for 96% of the total in actinobacteria. *Mycobacterium* was the most abundant genus, accounting for up to 22% of the total. Other rare actinobacteria, such as *Rubrobacter*, *Egicoccus*, *Ornithinicoccus*, *Rothia* and *Quadrisphaera*, were also dominant genera, in contrast to the predominant actinobacteria populations in the Atacama Desert (*Acidimicrobiaceae*, *Geodermatophilaceae* and *Micrococcaceae*). The culture results showed that actinobacteria were the most abundant bacteria in the soil of Flaming Mountain, with the highest proportion of 82%, which was comparable with reports from other “hyper-arid” areas of the world. We identified a total of 132 strains belonging to eight families and 11 genera, among which *Streptomyces* was the predominant genus (58%). Generally speaking, *Streptomyces* spp. are the main cultivable actinobacteria in arid areas. In the Atacama Desert, isolates in *Streptomyces* accounted for 76% of culturable actinobacteria [27]. In the Badain Jaran Desert, *Streptomyces* was the dominant genus (25%) [29]. Although rare actinobacteria were abundant in high-throughput sequencing, a few rare actinobacteria were identified among culturable strains [29]. In this work, 57 rare actinobacteria isolates were obtained (42%), which also proved the diversity of actinobacteria in the study area. Nocardiopsaceae was the most abundant family (12%), but only the genus *Nocardiopsis* was isolated from this culturable strain, which was the main culturable actinobacteria in the Sahara Desert [34]. Pseudonocardiaceae, which accounted for 12% in this work, were predominant in the Cholistan Desert [35]. The isolates belonging to Pseudonocardiaceae involved the most genera, including *Saccharomonospora*, *Pseudonocardia*, *Amycolatopsis* and *Actinokineospora*. Among them, *Amycolatopsis* and *Saccharomonospora* were also major genera in other hyper-arid environments [27,34], with *Amycolatopsis* in particular accounting for 17% of the culturable actinobacteria [27]. Additionally, only two strains were identified as *Blastococcus agglutatus*, which was firstly isolated from the surface of marble and limestone [36], whereas *Blastococcus* was dominant among the genera in the Tengger Desert, accounting for 18% of isolates [29]. However, there were far fewer culturable actinobacteria species than one might expect from the results of high-throughput sequencing.

The limiting factors of microbial growth in extreme environments are complex [37]. Due to the unsuitable growth environment [38], dormancy [39], and lack of population interaction [40], it is not possible to obtain all pure cultures of environmental microorganisms in the laboratory by conventional methods [41]. It is necessary to obtain more environmental microorganisms by simulating the environment, designing selective media and increasing the screening cycle. In this study, we used the Known Media Database (KO·MODO) (http://komodo.Modelseed.org/default.htm) [11] combined with environmental factors to design and optimize nine media (R2A and MA medium were designed on the basis of soil characteristics), which yielded 132 strains of actinobacteria. The most common medium for actinobacteria isolation was GAUZE’s medium, from which 17 strains of *Streptomyces* and 12 strains of rare actinobacteria were isolated. R2A medium had better selectivity to isolate rare actinobacteria, including *Georgenia*, *Nocardiopsis*, *Isoptericola* and *Saccharomonospora*. In addition, only rare species of actinobacteria were isolated using M3 medium. Notably, *Georgenia*, *Blastococcus* and *Amycolatopsis* were not detected by high-throughput sequencing, but were isolated on R2A, M9 and 656 media. In the past, the selection of isolation and screen media was usually based on empirical knowledge and literature reviews. Researchers often required a great deal of relevant knowledge and spent significant amounts of time. This database makes it more convenient and faster to select media, especially for junior experimenters. The media we selected were preferentially recommended by the database, and are generally considered as common media, although GAUZE’s and ISP2 medium were rarely reported in the isolation of common actinobacteria [42,43]. In addition, some uncommon media were recommended based on sequences in the database. In further study, we plan to include more uncommon media for actinobacterial culture to improve the isolation method. Notably, six strains were identified as potential novel bacterial species, with 16S rRNA gene similarity <98.65%. The diversity of culturable actinobacteria is lower than the results of high-throughput sequencing, showing that there are abundant actinobacteria resources on Flaming Mountain to be further explored. The culture results further supplemented the diversity of microbiota, which not only indicated that the high-throughput sequencing results had a certain deviation, but also provided valuable information for screening of microorganisms.

The extremely arid environment of the Turpan area is also conducive to the growth of environmental microorganisms with high temperature resistance, salt resistance, and other stress resistance characteristics. The results of strain characterizations showed that 57% of the strains could tolerate 5% NaCl, 85% of the strains could grow at pH 11.0, 79% of the strains could grow at 45 °C, and 9% of the strains could even grow at 55 °C. At the same time, it was found that actinobacteria in this hyper-arid region had strong enzyme production abilities. Li et al. screened a high-temperature protease-producing strain from the Turpan area [44], which broadened the resources for thermostable protease production. In the current study, 45% of the strains had amylase activity, and 52% had protease activity. In addition to enzymes, special environmental actinobacteria are also a treasure trove of natural compounds. For example, marine *Streptomyces* isolated from coastal areas of Vietnam produced furan compounds, which exhibited anti-proliferative activity against various types of cancer cells [45]. New compounds such as Ansamycin and a 22-member macrolactone with antibacterial and antitumor activities were obtained from the metabolites of actinobacteria isolated from the Atacama Desert [46]. It was found that the secondary metabolites of *Streptomyces* isolated from Taklimakan Desert contained Collismycins and other valuable antibacterial compounds [47]. The results of the study showed that 18% of the isolates had antimicrobial activity against *Escherichia coli*, 18% against *Pseudomonas aeruginosa*, 40% against *staphylococcus aureus*, 9% against *candida albicans*, and 8% against *Alternaria*, which may hold great significance for the research on novel microbiological drugs. At the same time, it was also found that 13% of the strains had quorum sensing inhibitory activity. In general, the bioactive molecules produced by heat and drought resistant actinobacteria are expected to have high thermal stability, bioavailability and solubility, which have obvious application advantages, laying a foundation for further research on the utilization of actinobacteria from arid areas.

## 5. Conclusions

The current work is the first report on the composition of the microbial community in the Flaming Mountain region. The results showed that a total of 22 phyla, 77 orders and 121 genera were identified by high-throughput sequencing, and actinobacteria were the dominant culturable bacteria (82%). A total of 132 strains of actinobacteria were identified, including eight families and 11 genera, among which six potential novel bacterial species were obtained. Further, most of the isolates showed high tolerance to saline-alkali and high temperature, and they could also produce amylase, protease, and cellulase. In addition, the metabolites of actinobacteria displayed obvious antimicrobial effects against common microbial pathogens, and 13% showed quorum sensing inhibition activity. Our results suggest that hyper-arid areas are a source for the isolation of actinobacteria that can produce a variety of enzymes and active secondary metabolites. However, further research is needed to explore the pharmaceutical and agricultural applications of actinobacteria.

## Figures and Tables

**Table 1:** Physical and chemical properties of soil samples from Flaming Mountain.

Sample Name	Location (Latitude, Longitude)	Soil Moisture (%)	pH	Organic Matter (g/kg)	Soluble Salt (g/kg)	Total Nitrogen (g/kg)
T1	42°92′98″ N, 89°52′54″ E	0.2	8.84	9.8	343.7	0.26
T2	42°93′40″ N, 89°55′40″ E	0.22	8.97	6.5	551	0.1
T3	42°93′64″ N, 89°53′12″ E	0.27	9.08	7.9	668.4	0.13
T4	42°93′47″ N, 89°53′62″ E	0.33	8.94	1.1	219.6	0.12
T5	42°94′51″ N, 89°52′12″ E	0.26	8.74	10	283.8	0.22

**Table 2:** Microbial count (CFU/g) by culture detection in Flaming Mountain samples.

Samples	CFU/g	CFU/g (Actinobacteria)	Actinobacteria Ratio (%)
T1	3.58 × 10^3^	2.54 × 10^3^	71
T2	2.1 × 10^3^	1.72 × 10^3^	82
T3	7.88 × 10^3^	6.14 × 10^3^	78
T4	2.47 × 10^3^	1.95 × 10^3^	79
T5	5.32 × 10^3^	4.25 × 10^3^	80

**Table 3:** Data statistics of genomic DNA sequences of soil samples.

Samples	Raw Number	Clean Number	Effective Number	OTU Number
T1	239,111	230,725	188,077	1690
T2	257,611	252,630	189,403	1157
T3	274,307	268,002	195,385	2762
T4	254,272	248,614	190,818	1611
T5	255,370	254,253	199,780	1226
Total	1,280,671	1,254,224	963,463	2994

**Table 4:** Classification of actinobacteria from Flaming Mountain region.

NO.	Family	Genus	Species	Number	Ratio (%)
1	Streptomycetaceae	*Streptomyces*	*S.thermolilacinus*	15	11.36%
			*S.werraensis*	2	1.51%
			*S.glomeratus*	4	3.03%
			*S.euryhalinus*	3	2.27%
			*S.pini*	2	1.51%
			*S.mutabilis*	1	0.75%
			*S.indiaensis*	6	4.54%
			*S.qinglanensis*	7	5.30%
			*S. nanhaiensis*	3	2.27%
			*S.rochei*	4	3.03%
			*S.radiopugnans*	12	9.09%
			*S.heliomycini*	6	4.54%
			*S.mangrovicola*	3	2.27%
			*S.lavendulocolor*	6	4.54%
			*S.kanasensis*	3	2.27%
2	Mycobacteriaceae	*Mycobacterium*	*M.arcueilense*	4	3.03%
3	Promicromonosporaceae	*Isoptericola*	*I.halotolerans*	2	1.51%
4	Microbacteriaceae	*Microbacterium*	*M.algeriense*	4	3.03%
			*M. oryzae*	2	1.51%
5	Nocardiopsaceae	*Nocardiopsis*	*N.dassonvillei*	16	12.12%
			*N.alborubida*	8	6.06%
6	Pseudonocardiaceae	*Saccharomonospora*	*S.azurea*	2	1.51%
		*Pseudonocardia*	*P.cypriaca*	3	2.27%
		*Amycolatopsis*	*A.marina*	7	5.30%
		*Actinokineospora*	*A.fastidiosa*	4	3.03%
7	Geodermatophilaceae	*Blastococcus*	*B.aggregatus*	2	1.51%
8	Micrococcales	*Georgenia*	*G.satyanarayanai*	1	0.75%

**Table 5:** Potential novel species based on 16S rRNA gene sequence analysis.

NO.	Similar Strain	Accession	Similarity
G24	*Streptomyces thermolilacinus*	NR 125444.1	98.35%
G36	*Saccharomonospora azurea*	NR 029371.1	98.07%
G85	*Streptomyces qinglanensis*	NR 109303.1	97.95%
G86	*Nocardiopsis dassonvillei*	NR 074635.1	98.32%
GH22	*Streptomyces pini*	NR 108264.1	98.08%
G150	*Georgenia satyanarayanai*	NR 117051.1	96.72%

**Table 6:** Resistance characteristics of partial respective isolates.

Name	Similar Strain	pH 12	pH 13	5% NaCl	10% NaCl	12% NaCl	45 °C	55 °C
G131	*Streptomyces indiaensis*	+	+	+	−	−	+	+
G86	*Nocardiopsis.* sp.	+	−	+	+	+	+	−
D119	*Streptomyces euryhalinus*	+	+	+	+	+	−	−
G24	*Streptomyces.* sp.	+	−	+	−	−	+	−
G114	*Streptomyces heliomycini*	+	+	+	+	−	+	+
D70	*Streptomyces mutabilis*	+	−	+	−	−	−	−
GH22	*Streptomyces.* sp.	+	−	+	−	−	+	−

Note: +: growth; −: no growth.

**Table 7:** Characteristics of the enzyme producing and antimicrobial activities and quorum sensing inhibitory activity of partial respective isolates.

NO.	Protease	Amylase	Cellulase	*E. coli*	*P. aeruginosa*	*S. aureus*	*C. albicans*	*A. alternata*	QSI Activity
G131	+	−	−	+	−	+	+	−	−
G86	+	+	−	−	+	+	−	−	−
D119	+	+	+	−	+	+	−	+	−
G24	−	+	−	−	+	−	+	−	+
G114	+	+	−	−	−	+	−	+	−
D70	−	+	−	+	−	−	+	−	−
GH22	+	+	−	−	−	+	−	−	−

Note: +: active; −: inactive.

## Data Availability

The OTU sequences have been deposited in GenBank of National Center for Biotechnology Information under the accession numbers ON810389-ON810472 and OP615608-OP615213, and the raw sequences in the Sequence Read Archive of NCBI under BioProject PRJNA923109.

## Supplementary Materials

The following supporting information can be downloaded at: https://www.mdpi.com/article/10.3390/microorganisms11020489/s1, Table S1: A total of 11 media were prepared to isolate actinobacteria.
