# Supplementary material for: Bacterial Community Composition and Isolation of Actinobacteria from the Soil of Flaming Mountain in Xinjiang, China
Source: Microorganisms. 2023 Feb 16;11(2):489. doi: 10.3390/microorganisms11020489 (PMC9967839; doi:10.3390/microorganisms11020489)
Supplement: Supplementary file 1 [file microorganisms-11-00489-s001.zip › microorganisms-2063429-supplementary.pdf]

Bacterial Community Composition and Isolation of Actinobacteria from the Soil of Flaming Mountain in Xinjiang, China

Zixuan He [1,2,†], Yuxian Wang [3,†], Xiaoyu Bai [4], Min Chu [2], Yuanyang Yi [2,4], Jing Zhu [2], Meiying Gu [2], Ling Jiang [3,*] and Zhidong Zhang [1,2,3,*]

1 College of Life Sciences and Technology, Xinjiang University, Urumqi 830046, China

2 Institute of Microbiology, Xinjiang Academy of Agricultural Sciences/Xinjiang Key Laboratory of Special Environmental Microbiology, Urumqi 830091, China

3 College of Food Science and Light Industry, State Key Laboratory of Materials-Oriented Chemical Engineering, Nanjing Tech University, Nanjing 211816, China

4 College of Life Sciences, Xinjiang Normal University, Urumqi 830054, China

* Correspondence: jiangling@njtech.edu.cn (L.J.); zhangzheedong@qq.com (Z.Z.)

† These authors contributed equally to this work.

*Corresponding authors.

Ling Jiang, Email: jiangling@njtech.edu.cn

College of Food Science and Light Industry, State Key Laboratory of Materials-Oriented Chemical Engineering, Nanjing Tech University, Nanjing 211816, China.

Zhidong Zhang, Email: zhangzheedong@sohu.com

Xinjiang Key Laboratory of Special Environmental Microbiology, Institute of Microbiology, Xinjiang Academy of Agricultural Sciences, Urumqi 830091, China

Supplementary Table S1. A total of 11 media were prepared to isolate Actinobacteria.

NO.	Medium	Medium formulation
1	GAUZE's Medium （g/L）	Starch soluble 20 g; KNO_3 1 g; $FeSO_4\cdot 7H_2O$ 0.01 g; $MgSO_4\cdot 7H_2O$ 0.5 g; Nacl 0.5 g; Agar 20 g ; H_2O 1 L; pH7.4-7.6
2	M2 medium（g/L）	Yeast Extract 4 g; Glucose 4 g; Malt extract powder 10 g; KNO_3 1 g; $FeSO_4\cdot 7H_2O$， 0.01 g; $MgSO_4\cdot 7H_2O$ 0.5 g; Agar 20 g; H_2O 1 L; pH7.8
3	M3 medium（g/L）	Starch soluble 20 g; KNO_3 2 g; k_2HPO_4 2 g; Cheesin 1 g; NaCl 2 g; $FeSO_4\cdot 7H_2O$， 0.01 g; $MgSO_4\cdot 7H_2O$ 0.5 g; $CaCO_3$ 0.02 g; Agar 20 g; H_2O 1 L
4	M6 medium（g/L）	Shrimp Shell 2 g; Humic acid 1 g; Na_2HPO_4 0.5 g; $MgSO_4\cdot 7H_2O$ 0.5 g; $FeSO_4\cdot 7H_2O$， 0.01 g; KCl 1.7 g; $CaCO_3$ 0.02 g; VB liquor (VB1 0.1 g ; Riboflavin 0.1 g; Niacin 0.1 g; VB_6 0.1 g; Inositol 0.1 g; Pantothenic acid 0.1g) 1ml; Agar 20 g; H_2O 1 L
5	M9 medium（g/L）	His 0.1 g ; KCl 1.7 g; D-Raffinose pentahydrate 1 g; $FeSO_4\cdot 7H_2O$， 0.01 g; $MgSO_4\cdot 7H_2O$ 0.5 g; $CaCO_3$ 0.02 g; Agar 20 g; H_2O 1 L

6	214 medium（g/L）	Yeast Extract 4 g; Glucose 4 g; Malt extract powder 10 g; $CaCO_3$ 2 g; Starch soluble 20 g; Agar 20 g; H_2O 1 L
7	R2A medium（g/L）	Yeast Extract 0.5 g; Peptone 0.5; Casein hydrolysate 0.5 g; Glucose 4 g; Starch soluble 0.5 g; Dipotassium hydrogen phosphate 0.3 g; Anhydrous magnesium sulfate 0.024 g; Sodium pyruvate 0.3 g; Agar 20 g; H_2O 1 L; pH7.2 ±0.2
8	656 medium（g/L）	Soluble starch 15 g; Yeast extract 4 g; K_2HPO_4 0.5 g; $MgSO_4 \cdot 7H_2O$ 0.5 g; Agar 15 g; H_2O 1 L; pH 7.4
9	SSY medium（g/L）	Yeast Extract 6 g; Glucose 5 g; KH_2PO_4 3g; MgSO4.7H_2O 0.5 g; $CaCl_2 \cdot 2H_2O$ 0.25 g; $(NH_4)_2SO_4$ 0.2 g; Trace elements($CaCl_2 \cdot$ $2H_2O$ 0.66 g; $CoCl_2$ •$6H_2O$ 0.18 g; $CuSO_4 \cdot 5H_2O$ 0.16 g; H_3BO_3 0.1 g) 1 mL; Agar 20 g; H_2O 1 L
10	MA medium（g/L）	Peptone 5 g; Yeast Extract 1 g; Nacl 19.45 g; $MgCl_2$ 5.98 g; Na_2SO_4 3.24 g; $C_6H_5FeO_7$ 0.1 g; $Cacl_2$ 1.8; KCl 0.55 g; Na_2CO_3 0.16 g; KBr 0.08 g; $SrCl_2$ 0.034 g; H_3BO_3 0.022 g; $Na_2O \cdot nSiO_2$ 0.004 g; NaF 0.0024 g; NH_4NO_3 0.0016 g; Na_2HPO_4 0.008 g; Agar 20 g; H_2O 1 L; pH 7.6
11	GSM medium（g/L）	Glucose 4 g; Yeast extract 4g; Malt extract 10 g; $CaCO_3$ 2 g; H_2O 1 L; Agar 20g; pH 7.2
